# Building positive workplace behaviors: how trust in the manager enhances sportsmanship behaviors through organizational trust

**DOI:** 10.3389/fpsyg.2026.1828581

**Published:** 2026-07-13

**Authors:** Hazem Aldabbas, Abdallah M. Elamin, Ahmed Z. E. Ahmed, Liza Gernal

**Affiliations:** College of Business Administration, Fujairah University, Fujairah, United Arab Emirates

**Keywords:** leader-member exchange (LMX), organizational citizenship behavior (OCB), organizational trust, social exchange theory (SET), sportsmanship behavior, trust in manager

## Abstract

Sportsmanship, a key dimension of organizational citizenship behavior (OCB), refers to employees’ willingness to maintain a positive attitude at work by tolerating inconveniences and avoiding unnecessary complaints. Although sportsmanship behavior plays an important role in sustaining cooperative and supportive workplace environments, relatively little research has examined the trust-related mechanisms that encourage it. Drawing on Social Exchange Theory (SET) and Leader-Member Exchange (LMX), this study examines how trust-based relationships within organizations influence employees’ sportsmanship behavior. Specifically, the study proposes that trust in the manager contributes to sportsmanship behavior both directly and indirectly through organizational trust. When employees perceive their managers as competent, fair, and supportive, they are more likely to interpret their interactions with managers as positive exchanges. According to SET, employees tend to reciprocate these positive experiences through constructive discretionary behaviors that benefit the organization. Over time, trust in the manager may extend beyond the interpersonal level and develop into a broader sense of trust in the organization. Data from 287 employees in Saudi Arabia were analyzed using PLS-SEM to examine how trust influences workplace behavior. The results indicate that when employees trust their manager, they are more likely to demonstrate sportsmanship behavior. Trust in the manager also strongly strengthens trust in the organization. In turn, organizational trust encourages sportsmanship behavior and partially explains how managerial trust translates into more positive behavior at work. Drawing on SET and LMX, the findings emphasize that building trustful manager–employee relationships can strengthen organizational trust and support a more cooperative, supportive work environment.

## 1 Introduction

Organizations depend not only on employees’ formal job performance but also on voluntary behaviors that extend beyond officially assigned duties. These discretionary actions, commonly referred to as Organizational Citizenship Behavior (OCB), play an important role in improving organizational effectiveness and supporting the overall functioning of the workplace (Organ, 1988). OCB reflects employees’ willingness to assist colleagues, sustain cooperation, and contribute positively to the organization in ways that may not be formally rewarded but remain vital to organizational success.

Among the dimensions of OCB, sportsmanship refers to employees’ willingness to tolerate less-than-ideal working conditions without excessive complaining or fault-finding (Podsakoff et al., 1990). Employees who demonstrate sportsmanship maintain a positive, constructive outlook even when facing difficult situations, helping organizations preserve a supportive, cooperative work environment. Sportsmanship behavior has become particularly relevant in modern organizational contexts characterized by uncertainty, rapid organizational change, and increasing performance expectations. Sportsmanship is the ability to maintain a positive attitude and refrain from excessive complaints when faced with workplace challenges, thereby contributing to organizational effectiveness (Blau et al., 2010). It also involves tolerating workplace inconveniences and unfavorable situations without complaining, thereby contributing to a positive work environment (Gupta, 2019).

Thus, employees who exhibit sportsmanship help maintain stability, encourage cooperation, and foster constructive engagement within organizations. In this regard, sportsmanship reflects employees’ positive workplace behavior, in which individuals focus on the constructive aspects of their organization and willingly accept unavoidable work-related inconveniences without complaining (Fernandes et al., 2023).

Organizational trust has long been regarded as a fundamental factor shaping positive workplace attitudes and behaviors (Hadler et al., 2025). Prior research consistently shows that trust in leadership plays a significant role in encouraging employees’ OCB and improving job performance (Dirks and Ferrin, 2002). Trust promotes cooperation, strengthens relationships between employees and their leaders, and motivates individuals to contribute beyond their formal job duties.

Drawing on Social Exchange Theory (SET) (Blau, 1964; Cropanzano and Mitchell, 2005) and Leader–Member Exchange (LMX) (Dansereau et al., 1975; Graen and Uhl-Bien, 1995), this study suggests that employees who trust their managers are more likely to develop a broader sense of trust in the organization as a whole, which, in turn, encourages them to display sportsmanship behaviors. SET posits that when employees perceive trust, fairness, and support from their leaders, they tend to reciprocate with positive attitudes and discretionary behaviors that benefit the organization. Accordingly, this study proposes that organizational trust mediates the relationship between trust in the manager and sportsmanship.

Although previous studies acknowledge the importance of organizational trust in encouraging OCB (Altuntas and Baykal, 2010; Kidron and Vinarski Peretz, 2026; Raziq et al., 2025; Singh and Srivastava, 2016), there remains limited agreement on the mechanisms through which this relationship operates. The mediating processes that link organizational trust to employees’ OCB remain insufficiently understood, highlighting an important gap that requires further theoretical and empirical investigation (Dai et al., 2022). Additionally, relatively little attention has been given to the underlying mechanisms through which trust in managers leads to specific forms of OCB, such as sportsmanship behavior. In particular, the role of organizational trust as a mediator in this relationship remains insufficiently examined.

This study examines how trust in managers influences organizational trust and positive sportsmanship behavior among employees in Saudi Arabia. Drawing on SET and trust literature, particularly Mayer et al.’s (1995) conceptualization of trust relationships, this study investigates the mediating role of organizational trust in explaining how managerial trust relationships may encourage positive discretionary workplace behaviors (sportsmanship behavior). Sportsmanship behavior, as a dimension of OCB, was specifically chosen because it reflects employees’ willingness to tolerate workplace inconvenience, avoid unnecessary complaints, and maintain positive attitudes within the organization.

These discretionary behaviors are closely linked to trust-based social exchange relationships and the positive relational mechanisms emphasized in Mayer et al. (1995) and SET. Compared with other OCB dimensions, sportsmanship is especially relevant to organizational trust contexts because it captures employees’ behavioral responses to supportive, trustworthy workplace relationships. Additionally, contemporary organizations increasingly face financial and operational challenges that require employees to remain adaptable, positive, and supportive in the workplace. Sportsmanship is considered particularly relevant because it reflects employees’ ability to tolerate workplace inconvenience, avoid exaggerating negative situations, and maintain constructive attitudes during challenging circumstances (Gupta, 2019).

Moreover, Saudi Arabia offers a distinctive setting for examining trust relationships and OCBs, given the ongoing economic transformation under Vision 2030, which prioritizes organizational effectiveness, employee engagement, and human capital development (Asfahani, 2025). The Saudi workforce reflects a blend of traditional collectivist values and hierarchical structures alongside increasing modernization. This blend creates a context in which trust in managers and organizations may influence employee behavior differently than in Western settings (Alotaibi and Campbell, 2022). Furthermore, research on sportsmanship as a component of OCB in Saudi Arabia remains limited (Elamin and Tlaiss, 2015). By investigating the mediating role of organizational trust between trust in managers and sportsmanship, our study contributes context-specific evidence and extends the trust literature beyond predominantly Western contexts.

Therefore, the present study addresses the following research question (RQ):

*RQ: How does trust in a manager promote organizational trust and positive sportsmanship behavior among employees in Saudi Arabia?*

This study contributes to the literature in several ways. First, it integrates SET to develop a conceptual model linking trust in manager, organizational trust, and sportsmanship. Second, it empirically examines the mediating role of organizational trust in explaining how managerial trust influences employees’ discretionary behaviors. Third, the findings provide practical insights for organizations seeking to foster constructive workplace attitudes by strengthening trust relationships between leaders and employees.

The remainder of this paper is organized as follows. The next section reviews the relevant literature and develops the theoretical framework and hypotheses. This is followed by the methodology and data analysis sections. The study then presents and discusses the findings, followed by the implications, limitations, and recommendations for future research.

The next section presents the theoretical background and develops the study hypotheses.

## 2 Literature review and theoretical background

### 2.1 Social exchange theory (SET) and leader-member exchange (LMX)

Given the dynamic and multifaceted nature of this environment, the assumptions of SET and the LMX perspective provide a useful lens for examining these relationships (March et al., 2026).

Social Exchange Theory (SET) explains social relationships as reciprocal exchanges of valued resources (Blau, 1964). When individuals receive favorable treatment, they feel obligated to reciprocate (Cropanzano and Mitchell, 2005). SET also posits that reciprocal exchanges between employees and organizations generate mutual benefits that foster trust and commitment in the workplace (Aldabbas et al., 2025). In organizational settings, employees interpret fairness, support, and integrity as social currency that shapes their attitudes and behaviors.

SET has been widely used to explain citizenship behaviors, employee engagement, and trust-based workplace outcomes (Cropanzano and Mitchell, 2005; Eisenberger et al., 1986). A social exchange relationship is built on mutual trust and develops through ongoing interactions between individuals. It involves long-term commitments and shared expectations, with the exchange often centering on socio-emotional elements such as trust, obligation, and commitment (Aldabbas and Blaique, 2025; Kuvaas et al., 2020).

LMX theory explains leadership through the quality of one-to-one relationships between leaders and followers (Dansereau et al., 1975). Hence, LMX theory posits that the quality of leader–member interactions represents a core organizational mechanism through which collective performance, coordination, and effectiveness are influenced (March et al., 2026). LMX theory suggests that leaders establish relationships of varying quality with different employees, leading to differences in workplace attitudes and outcomes. High-quality LMX relationships are typically marked by trust, mutual respect, and reciprocal resource exchange (Willie, 2025).

Mayer et al. (1995) proposed one of the most influential frameworks for organizational trust, defining trust as a party’s willingness to be vulnerable to another party’s actions based on positive expectations about that party’s behavior. The model emphasizes that trust develops through perceptions of ability, benevolence, and integrity, which in turn influence relational outcomes and risk-taking behaviors within organizations. In organizational settings, trusted relationships between employees and managers may encourage employees to engage in discretionary behaviors that go beyond formal job requirements. Accordingly, building on SET, the present study extends this perspective by examining how trust in managers contributes to organizational trust and subsequently enhances sportsmanship behavior as a dimension of OCB.

### 2.2 Trust in manager

Trust is the willingness to be vulnerable based on positive expectations about another party’s intentions or behavior (Mayer et al., 1995). Trust in the manager refers specifically to employees’ confidence in their immediate supervisor’s competence, integrity, and benevolence (McAllister, 1995). Meta-analytic evidence shows that trust in leadership significantly predicts citizenship behaviors and performance outcomes (Dirks and Ferrin, 2002). When employees trust their managers, they reduce defensive behaviors and increase cooperative engagement. Managers serve as agents of the organization. Therefore, employees often interpret managerial actions as reflections of organizational values (Rousseau et al., 1998).

Recent research underscores the critical importance of managerial trust in both sports and organizational contexts. For example, a study of college sports environments found that athletes’ trust in their coaches increased significantly when they perceived the procedures as fair. This, in turn, increased their commitment to their teams and their likelihood of feeling supported by their organizations (POS). These results confirm the importance of leader fairness and open decision-making in building trust (Han and Ha, 2025).

Leaders who show charisma, care about each person, and challenge their minds build more trust among their employees, which in turn encourages positive behaviors such as sportsmanship. Trust functions as a psychological mechanism through which leadership style affects employees’ discretionary behavior (Johnsen, 2026).

### 2.3 Organizational trust

Organizational trust is employees’ confidence in the organization’s fairness, reliability, and integrity (Cook and Wall, 1980). It reflects trust in the organization as a system rather than in an individual. Research shows that organizational trust predicts positive workplace outcomes, including commitment, engagement, and OCB (Colquitt et al., 2007; Dirks and Ferrin, 2002). Trust transfer theory holds that trust in a specific actor (e.g., a manager) can generalize to a broader entity (e.g., the organization) (Rousseau et al., 1998).

Recent studies across sectors confirm the mediating role of organizational trust: an empirical study of referees found that organizational trust partially mediated the link between perceived organizational support and job satisfaction and fully mediated the link between support and career commitment (Liu et al., 2025). These findings collectively support SET; when employees perceive support and equitable treatment, they cultivate trust in their organizations, which subsequently improves job satisfaction, commitment, and performance (Cropanzano and Rupp, 2008). Additionally, an empirical study of Chinese companies showed that trust, when combined with strong support from supervisors and clan-oriented cultures, was a strong predictor of employees’ emotional commitment. This indicates that the influence of trust is contingent on context and is enhanced by supportive cultural characteristics (Zhang and Gao, 2025).

### 2.4 Sportsmanship behavior

Sportsmanship behavior is defined as employees’ willingness to tolerate inconvenience without excessive complaining and the maintenance of a positive attitude (Nyarieko, 2018; Podsakoff et al., 1990). Sportsmanship behavior extends beyond the workplace and contributes significantly to the development of responsible and constructive citizenship (Çitil and Karayol, 2022). Furthermore, sportsmanship is characterized by maintaining optimism and patience in the face of workplace challenges, difficulties, or unfavorable conditions (Özdemir and Büyükyılmaz, 2025).

In Saudi workplaces, sportsmanship is a crucial component of OCB because it aligns with the nation’s social norms, cultural customs, and religious beliefs (Ababneh et al., 2026). Islamic teachings encourage patience, tolerance, forgiveness, cooperation, and good relationships, so employees are expected to handle workplace challenges calmly and put the group’s needs ahead of their own. Saudi culture values collaboration, respect for authority, and strong relationships, so sportsmanship helps maintain a peaceful workplace, reduce conflict, and support teamwork (Alkahtani et al., 2021). Employees who demonstrate sportsmanship help create a constructive work environment by facing challenges, adapting to change, and staying positive during difficult times (Yang et al., 2023). Socially, Saudi workplaces value respect, unity, and relationship-building, and positive behavior fosters trust and teamwork (Alotaibi and Campbell, 2022). Because of this, sportsmanship not only improves how people work together but also strengthens and unites organizations, which is especially important in Saudi Arabia (Elamin and Tlaiss, 2015).

In leadership contexts, recent research shows that sportsmanship is strongly influenced by trust in leaders. A study found that trust in leadership significantly predicted employees’ sportsmanship, while trait emotional intelligence partially mediated this relationship. This suggests that trust not only promotes conformity but also advances positive, flexible behavior (Gupta, 2019). Organ (1988) conceptualized sportsmanship as a key dimension of OCB, alongside altruism, courtesy, conscientiousness, and civic virtue. Employees who demonstrate sportsmanship maintain positive attitudes even when facing organizational constraints. This behavior reduces workplace negativity and enhances collective functioning.

Sportsmanship behavior has been found to increase employee commitment in service-oriented industries. In a study conducted in Nigeria’s hospitality industry during the 2023–2024 period, a positive correlation was found between the manager’s sportsmanship behavior and the employee’s affective and normative commitment. This shows that when the manager acts ethically and treats the employee respectfully and positively, it will have a significant impact on the employee’s commitment (Ejumudo et al., 2024).

## 3 Hypotheses development

### 3.1 Trust in manager and organizational trust

Trust in managers is widely recognized as a fundamental aspect of organizational life because it influences employees’ attitudes, behaviors, and workplace relationships (Trivedi et al., 2010). Thus, trust in managers can be understood as employees’ positive expectations regarding the intentions and future actions of their supervisors, particularly the belief that such actions will be beneficial rather than detrimental to their wellbeing (Robinson, 1996). Organizational trust is defined as “a psychological state comprising willingness to accept vulnerability based on positive expectations of an organization” (Fulmer and Gelfand, 2012, p. 1174). Organizational trust plays a central role in social exchange relationships, as employees often rely on their interactions with managers to form broader perceptions of the organization (Liu et al., 2025). When managers demonstrate trustworthiness, support, and fairness, employees are more likely to develop confidence not only in their supervisors but also in the organization they represent.

Evidence from the sports industry shows that trust in the manager (coaches, referees’ supervisors) directly affects employees’ organizational trust, which, in turn, directly affects attitudinal outcomes such as job satisfaction and commitment. This pattern is consistent with SET and LMX theory (Liu et al., 2025). When managers demonstrate fairness, competence, and integrity, employees infer that the organization supports such behavior (Rousseau et al., 1998).

Although perceived organizational support and motivation to volunteer have been identified as important antecedents of OCB (Kao et al., 2023), prior research suggests that trust-related factors may also play a critical role in encouraging discretionary workplace behaviors. Organizational trust is grounded in employees’ rational assessments of others’ trustworthiness, which develop through accumulated experience, interactions, and knowledge of organizational members’ capabilities and reliability (Pucetaite and Novelskaite, 2014).

When employees trust their managers and the organization and perceive a supportive, effective work environment, they are more likely to reciprocate with discretionary positive behaviors that go beyond formal job requirements. Therefore, it is reasonable to expect that higher levels of trust in managers will contribute to the development of organizational trust among employees. Because managers act as key organizational representatives, employees who perceive their immediate supervisors as trustworthy are more likely to extend that trust to the organization itself, resulting in higher levels of organizational trust (Legood et al., 2016). Based on the previous argument, we formulate the first hypothesis:

*H1*: Trust in manager positively influences organizational trust.

### 3.2 Organizational trust and sportsmanship behavior

Organizational trust also plays a key role in shaping positive workplace behaviors. When employees trust their organization, they are more likely to cooperate with colleagues, support organizational goals, and contribute beyond their formal job responsibilities. This trust strengthens constructive, collaborative behaviors essential to effective teamwork and a healthy organizational climate (Dirks and Ferrin, 2002). Saks and Gruman (2024) argued that organizational trust reflects employees’ confidence in the organization’s ability, benevolence, and integrity, and develops when employees believe the organization genuinely cares about their wellbeing and acts in their best interests.

Previous trust research has emphasized that trust relationships within organizations develop through positive social exchange and may subsequently influence broader organizational outcomes (Schaubroeck et al., 2013). Building on Mayer et al. (1995) and Schaubroeck et al. (2013) argued that leaders often represent the organization and its values, so positive trust relationships with leaders may extend to broader organizational trust perceptions. Consequently, employees who trust their managers may develop stronger organizational trust, which can encourage positive workplace behaviors such as sportsmanship.

When employees trust their organization, they are more likely to interpret workplace challenges in a balanced, constructive way. Rather than viewing difficulties as signs of unfair treatment or exploitation, employees tend to see them as normal or temporary situations that can occur in any work environment. This perception helps reduce negative reactions such as cynicism, frustration, and excessive complaining (Colquitt et al., 2007). Trust in the organization creates a psychological foundation in which employees believe that management decisions are made fairly and with good intentions, encouraging them to remain patient and cooperative even during demanding periods.

Organizational trust is fostered by shared organizational values, beliefs, and behavioral practices, creating an environment that facilitates open communication and positive interactions among employees (Ertosun and Aşçı, 2021). Thus, sportsmanship is considered a voluntary, discretionary workplace behavior that goes beyond employees’ formal job responsibilities (Silva et al., 2023). It reflects employees’ willingness to maintain positive attitudes, tolerate workplace inconveniences, and avoid unnecessary complaints, even in challenging situations. SET suggests that employees who experience fair and supportive treatment from their organizations reciprocate by engaging in discretionary behaviors such as sportsmanship (Cropanzano and Mitchell, 2005). Based on the previous arguments, we formulate the following hypothesis:

*H2*: Organizational trust positively influences sportsmanship behavior.

### 3.3 Trust in manager and sportsmanship behavior

Fairness, managerial support, and principled leadership create a positive organizational climate where trust can flourish. This trust fosters higher levels of commitment, encourages constructive behaviors, and reduces disengagement and negative attitudes among employees (Akar, 2018). When employees believe that their manager is willing to consider and accommodate their personal needs, such as allowing extra break time during urgent family situations, they often interpret this behavior as a sign of support, understanding, and fairness within the workplace (Alnagbi et al., 2025).

Such actions signal that the manager values employees not only as workers but also as individuals with personal responsibilities. According to SET, when managers provide this type of discretionary support, employees tend to respond with positive attitudes and behaviors (Colquitt and Rodell, 2011). One form of such positive workplace behavior is sportsmanship. Supportive treatment strengthens trust between employees and their supervisors and fosters a sense of reciprocal obligation. As a result, employees become more motivated to contribute beyond their formal job requirements, demonstrating greater engagement and participating in OCBs that benefit the workplace.

When employees consistently observe supportive managerial behaviors and fair interpersonal treatment, they begin to extend this trust beyond individual supervisors to the organization as a whole. Over time, this process strengthens employees’ emotional attachment to their workplace and increases their willingness to maintain positive attitudes even in challenging situations. Consequently, employees who feel trusted and respected are more likely to engage in constructive behaviors, cooperate with colleagues, and contribute to a stable, supportive organizational environment. Thus, when workers perceive high service quality and trust their manager, they are more likely to display discretionary positive behavior, such as sportsmanship (Kim et al., 2019). Specifically, when employees perceive their managers as fair, supportive, and trustworthy, they are more likely to develop positive attitudes toward the workplace and feel secure in expressing their views and concerns (Assefa et al., 2024). Furthermore, when employees trust their leaders, they are more likely to maintain positive attitudes, tolerate minor inconveniences, and avoid unnecessary complaints. Thus, we formulate the third hypothesis:

*H3*: Trust in manager positively influences sportsmanship behavior.

### 3.4 the mediating role of organizational trust

Organizations that strengthen organizational trust through supportive, trustworthy managerial relationships are more likely to cultivate committed, cooperative employees (Klimchak et al., 2020). When employees trust their managers, they may perceive the organization as fair, reliable, and supportive, leading them to generalize this trust to the broader organization. Consequently, employees with higher organizational trust are more likely to demonstrate sportsmanship by tolerating workplace inconveniences, avoiding unnecessary complaints, and maintaining positive, cooperative attitudes in their daily work (Özdemir and Büyükyılmaz, 2025).

According to SET and LMX theory, employees who experience supportive, trusting relationships with their supervisors are more likely to maintain positive, reciprocal relationships within the organization (Badru et al., 2024). High-quality manager–employee relationships, characterized by trust, mutual respect, and effective communication, may strengthen employees’ positive perceptions of the organization and encourage constructive workplace behaviors. Consequently, employees who develop stronger organizational trust are more likely to demonstrate sportsmanship through cooperation, tolerance, and fewer workplace complaints (Podsakoff et al., 2000).

Mayer et al. (1995) argued that trust encourages individuals to engage in relationship-based risk-taking within organizations. Such behaviors may include discretionary and extra-role actions that support organizational functioning. In this context, sportsmanship reflects employees’ willingness to tolerate inconvenience, avoid unnecessary complaints, and maintain positive attitudes that support organizational effectiveness. Therefore, employees with higher organizational trust may be more likely to demonstrate sportsmanship.

SET further explains this process by suggesting that positive exchanges between individuals create expectations of reciprocity (Blau, 1964). When employees perceive their relationship with their manager as supportive and trustworthy, they view it as a high-quality exchange relationship. Over time, this trust can extend from the manager to the organization, encouraging employees to maintain positive attitudes and behaviors at work. Trust in managers has been shown to directly influence sportsmanship behavior by encouraging employees to go beyond formal job responsibilities and maintain constructive attitudes. When employees believe that their managers act with good intentions and make fair decisions, they are more likely to reduce complaint behavior and demonstrate tolerance toward workplace challenges (Gullu and Yildiz, 2019; Özdemir and Büyükyılmaz, 2025). Furthermore, when employees perceive that their organization provides support through constructive feedback and a supportive work environment, they are more likely to develop trust in the organization. This trust, in turn, encourages employees to engage in voluntary behaviors that contribute to organizational sustainability and support the satisfaction of external stakeholders (Park and Kim, 2024). According to LMX theory, effective leader–member relationships are built on trust, mutual respect, and open communication (Assefa et al., 2024; Graen and Uhl-Bien, 1995). Thus, we formulate the last hypothesis:

*H4*: Organizational trust mediates the relationship between trust in the manager and sportsmanship behavior.

**FIGURE 1:**
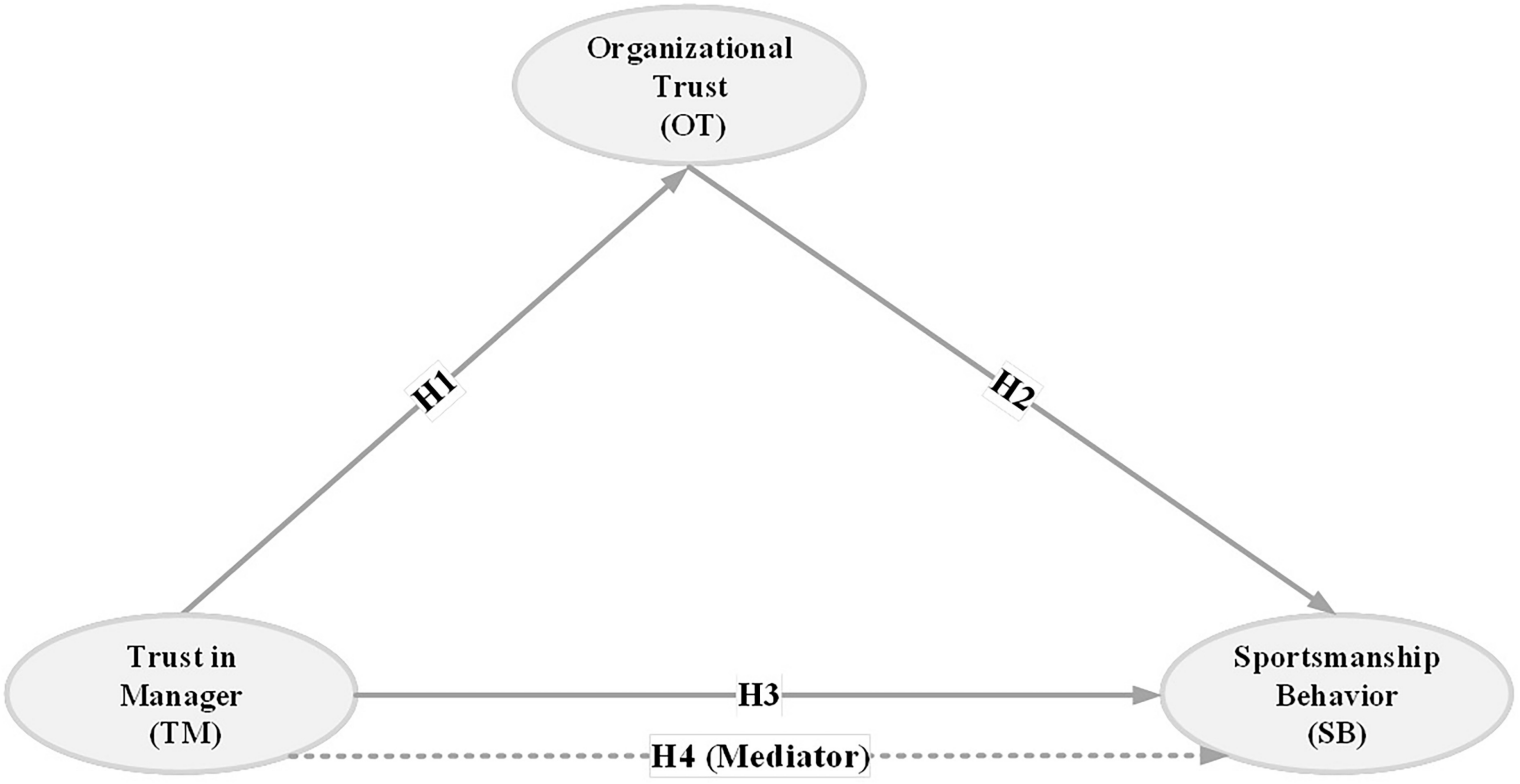
Hypothesized framework.

The proposed research model is presented in Figure 1.

## 4 Methodology

### 4.1 Sample

The questionnaire was constructed using previously validated scales widely used in organizational behavior research. Items assessing trust in the manager, organizational trust, and sportsmanship were adapted from established instruments to ensure content validity. Prior to the main data collection, we conducted a pilot study with 30 MBA students employed in organizations similar to those in the final sample. Participants evaluated item clarity, wording, relevance, and comprehensibility. Based on their feedback, we made minor adjustments to improve readability and cultural appropriateness. The pilot study demonstrated satisfactory internal consistency, with Cronbach’s alpha values above the recommended threshold of 0.70 for all constructs (Al-Haroon and Al-Qahtani, 2020). These steps enhanced the instrument’s reliability and confirmed the questionnaire’s suitability for the Saudi organizational context before large-scale administration.

To examine sportsmanship as a dimension of OCB, along with employees’ perceptions of organizational trust and trust in their direct supervisors, a structured self-report survey was designed to collect the required data. A self-administered questionnaire was chosen because the key variables in this study relate to employees’ perceptions and attitudes. Conducting organizational research in the Middle East often involves methodological challenges, including limited organizational access, cultural sensitivities, and a general hesitation to participate in survey-based studies (Elamin, 2024). Given these constraints, convenience sampling was adopted (Emerson, 2021). Although this method may limit statistical generalizability, it is commonly used and considered practical when probability sampling is difficult to implement (Polas, 2024).

Formal invitations were sent to Human Resources departments at organizations across industries in Riyadh, the capital of the Kingdom of Saudi Arabia, and in the Western Province. Nine organizations agreed to participate and granted access to their employees. Organizational approval was obtained, and strict assurances of confidentiality and anonymity were provided to both the organizations and the respondents. Participation was voluntary, and all respondents were informed of the study’s purpose and provided written informed consent before completing the survey. These procedures were implemented to ensure ethical research practices and to encourage participant trust.

To facilitate participation and accommodate organizational preferences, the questionnaire was distributed online. Participating organizations shared the survey link electronically with their employees, enabling respondents to complete and submit the questionnaire conveniently while maintaining confidentiality.

In total, 600 questionnaires were distributed, and 302 responses were received, yielding a response rate of 47.8%, which is considered acceptable for organizational research in this regional context. After screening for completeness, consistency, and response quality, 15 questionnaires were excluded due to missing or inconsistent responses. For surveys with minimal missing data, mean substitution was applied where appropriate. Consequently, 287 valid questionnaires were retained for the final analysis, ensuring a reliable dataset for subsequent statistical procedures.

Table 1 presents the demographic characteristics of the sample (*N* = 287). Regarding nationality, 154 respondents (53.7%) were Saudi, and 133 (46.3%) were non-Saudi, indicating a balanced representation of local and expatriate employees. The sample was predominantly male (95.1%), with female respondents comprising 4.9%, which may reflect the workforce composition in certain industries within the region.

**TABLE 1:** Demographics of the sample.

Variables	Frequency	Percentage (%)
Nationality		
Saudi	154	53.7
Non-Saudi	133	46.3
Gender		
Male	273	95.1
Female	14	4.9
Age		
35 years or less	166	57.8
36–46 years	88	30.7
47 years or above	33	11.5
Educational level		
High school or below	85	29.6
Diploma	87	30.3
Bachelor or above	115	40.1
employment sector		
Private	234	81.5
Public	53	18.5
Job tenure		
5 years or less	133	46.3
6–10 years	91	31.7
11 years or more	63	22
Organizational tenure		
5 years or less	98	34.1
6–10 years	65	22.6
11 years or more	124	43.2

By age, most respondents were 35 or younger (57.8%), followed by those aged 36–46 (30.7%) and those 47 or older (11.5%), suggesting the sample largely consisted of early- to mid-career professionals. In terms of education, 40.1% held a bachelor’s degree or higher, 30.3% had a diploma, and 29.6% had a high school certificate or lower, indicating a moderately educated workforce.

The majority of respondents were employed in the private sector (81.5%), while 18.5% worked in the public sector. Participants represented several industries, including manufacturing (33.8%), food and beverage (21.9%), engineering and construction (19.9%), education (8.5%), and telecommunications and technology (5.9%). This sectoral diversity strengthens the study’s external validity by capturing perspectives from multiple organizational contexts.

Regarding work experience, 46.3% of respondents had five years or less of professional experience, 31.7% had between 6 and 10 years, and 22.0% had 11 years or more. In terms of organizational tenure, 43.2% had more than 11 years in their organizations, 34.1% had 5 years or less, and 22.6% had between 6 and 10 years.

Overall, the sample reflects a predominantly male, private-sector workforce composed mainly of early- to mid-career employees from diverse industries and educational backgrounds. This diversity strengthens the empirical analysis and supports the broader applicability of the study’s findings across organizational settings.

Overall, the sample reflects a predominantly male, private-sector workforce composed mainly of early- to mid-career employees from diverse industries and educational backgrounds. Specifically, 95.1% of respondents were male, while only 4.9% were female. Although the sample demonstrates diversity in industry, experience, and education, the gender distribution was highly skewed toward male respondents. However, a one-way ANOVA revealed no significant differences between male and female respondents in organizational trust (*F* = 0.759, *p* = 0.384) or sportsmanship (*F* = 0.615, *p* = 0.434), suggesting that gender did not significantly influence the study variables. Nevertheless, caution should be exercised when generalizing the findings to populations with a more balanced gender composition.

### 4.2 Measures

The conceptualization of trust in this study was informed by Mayer et al. (1995), who viewed trust as a relational mechanism associated with vulnerability, positive expectations, and workplace behavioral outcomes. Based on this perspective, trust in the manager and organizational trust were treated as distinct but related constructs within the organizational context. This conceptual distinction supports examining how managerial trust relationships may influence broader organizational trust perceptions and, in turn, shape employees’ discretionary workplace behaviors, particularly sportsmanship. Given the exploratory nature of this study, all constructs were measured using well-established, previously validated scales to ensure conceptual clarity, consistency with prior research, and methodological rigor.

Trust was examined at two related levels: trust in the immediate supervisor (trust in the manager) and trust in the organization. *Trust in the manager* was measured using the six-item scale developed by Podsakoff et al. (1990), which assesses the extent to which employees perceive their supervisors as fair, reliable, and supportive.

*Organizational trust* was measured with Robinson’s (1996) seven-item scale, which assesses employees’ overall confidence in the organization’s integrity, fairness, and fulfillment of its obligations. Using these established instruments strengthens construct validity and aligns with prior empirical studies in organizational behavior and management research. The full scales for trust in the manager and organizational trust are provided in Appendix 1.

*Sportsmanship*, conceptualized as a dimension of OCB, was measured using the scale originally developed by Niehoff and Moorman (1993) and later adapted by Desivilya et al. (2006) to assess managers’ citizenship behaviors. The original instrument covers five classical OCB dimensions, including five items that specifically capture sportsmanship. This dimension reflects employees’ willingness to tolerate minor inconveniences and workplace frustrations without excessive complaining, thereby helping maintain a positive organizational climate. The full sportsmanship scale is presented in Appendix 1.

Following Kabasakal et al. (2011), all measures were adapted to a self-report format, which is appropriate for capturing perceptual and attitudinal constructs. These constructs are best assessed through individuals’ own evaluations of their experiences and relationships in the workplace. All items were measured using a five-point Likert-type scale ranging from 1 (“Never”) to 5 (“Always”), allowing respondents to indicate the frequency of the described behaviors or perceptions.

### 4.3 Procedures

Data were collected using a structured survey questionnaire designed to ensure clarity, cultural appropriateness, and methodological rigor. The questionnaire consisted of four main sections. The first section gathered demographic and organizational information, including nationality, gender, age, educational level, employment sector, job tenure, and organizational tenure. These variables described the sample’s characteristics and provided useful context for subsequent comparative and control analyses. The remaining sections measured the study’s key constructs: trust in the immediate supervisor, trust in the organization (employer), and sportsmanship as a dimension of OCB. All scales were presented in a consistent, standardized format to facilitate completion and reduce respondent fatigue.

The online survey method was particularly appropriate for the Saudi organizational context for several reasons. First, organizations are geographically dispersed across major regions, including Riyadh and the Western Province, making electronic distribution practical and cost-effective. Second, online surveys offer greater anonymity and confidentiality, which are crucial in high power-distance cultures where employees may hesitate to share perceptions of supervisors and organizations in person (Mertens and Schollaert, 2024). Third, the increasing digitalization of organizational processes and the widespread use of electronic communication platforms in Saudi Arabia have increased employees’ familiarity with online surveys, facilitating participation and improving response accuracy (Daikeler et al., 2022). Finally, online administration allowed respondents to complete the questionnaire at their convenience, reducing social desirability bias and encouraging more candid responses regarding trust and organizational behaviors. Thus, the online survey method was both culturally and methodologically appropriate for data collection in the Saudi context.

Because Arabic is the official language of the Kingdom of Saudi Arabia (KSA), distributing the survey only in English could have limited participants’ understanding and potentially affected response quality and participation rates. To ensure linguistic accuracy and cultural appropriateness, a back-translation procedure was used. Following established methodological guidelines (e.g., Elamin, 2024), two independent bilingual translators first translated the original English questionnaire into Arabic using a conceptually accurate approach. Two other translators, who had not seen the original questionnaire, then translated the Arabic version back into English.

The research team, together with language professors fluent in Arabic and English, compared the original English questionnaire with the back-translated version. Minor differences were discussed and corrected to ensure conceptual equivalence and grammatical clarity without altering the meaning of the items. Most adjustments were linguistic and did not affect the original validated scales. To further ensure clarity and face validity, the final Arabic questionnaire was pilot tested with a small group of students who provided feedback on wording and overall clarity. The pilot test indicated that the questionnaire was clear and easy to understand, so no further changes were required.

These procedures helped ensure linguistic accuracy, cultural sensitivity, and measurement equivalence, thereby strengthening the reliability and validity of the data collection process. Data collection was conducted after obtaining ethical approval from the Research Ethics Committee under approval number UOF/REC/2025-02-15.

### 4.4 Results

Before assessing the measurement model, the data’s suitability for factor analysis was evaluated. The Kaiser-Meyer-Olkin (KMO) measure of sampling adequacy was 0.807, exceeding the recommended threshold of 0.60 (Shrestha, 2021). Bartlett’s Test of Sphericity was statistically significant (χ^2^ = 2450.316, df = 153, *p* < 0.001), indicating that the correlation matrix was appropriate for factor analysis. The reproduced correlation matrix was also examined to assess the adequacy of the factor structure and the extent to which the extracted factors reproduced the observed correlations. These results confirmed the data’s suitability for subsequent measurement model evaluation.

#### 4.4.1 Measurement model assessment

To assess whether the measurement model was acceptable, we conducted the standard reliability and validity tests recommended for PLS-SEM (Hair et al., 2019). Table 2 presents the indicator loadings. All items we retained had loadings above the 0.50 cutoff, indicating acceptable indicator reliability. Items with loadings below 0.50 were removed to improve the model: three Organizational Trust items (OT1, OT2, OT4) and two Trust in Manager items (TM4, TM5) were dropped for low loadings.

**TABLE 2:** Factor loadings.

Main variables	Organizational trust (OT)	Sportsmanship behavior (SB)	Trust in manager (TM)
OT3	0.824		
OT5	0.827		
OT6	0.523		
OT7	0.772		
SB1		0.838	
SB2		0.842	
SB3		0.885	
SB4		0.879	
SB5		0.776	
TM1			0.762
TM2			0.563
TM3			0.745
TM6			0.770

The study’s reliability and convergent validity results are shown in Table 3. Cronbach’s alpha (CA) and composite reliability (CR) were used to assess reliability. The results demonstrate that the measurement items have acceptable internal consistency, with Cronbach’s alpha values ranging from 0.736 to 0.899, all of which are higher than the suggested cutoff of 0.70 (Hair et al., 2019). Similarly, the composite reliability (CR) values are significantly higher than the suggested minimum level of 0.70, ranging from 0.899 to 0.926. This provides additional evidence for the validity of the study’s constructs.

**TABLE 3:** Cronbach’s alpha, composite reliability, AVE.

Main variables	Cronbach’s alpha	Composite reliability (rho_c)	Average variance extracted (AVE)
Organizational trust (OT)	0.736	0.831	0.558
Sportsmanship behavior (SB)	0.899	0.926	0.714
Trust in manager (TM)	0.736	0.899	0.511

The Average Variance Extracted (AVE) was used to assess convergent validity. All AVE values, ranging from 0.511 to 0.714, exceed the recommended threshold of 0.50. This indicates that more than half of the variance in each indicator is explained by the constructs. Overall, these findings show that the measurement scales exhibit adequate convergent validity and satisfactory reliability, indicating that the constructs are appropriate for further structural model analysis.

#### 4.4.2 Multicollinearity assessment

The Variance Inflation Factor (VIF) was used to assess multicollinearity among the predictor constructs before testing the structural relationships. According to Hair et al. (2019), VIF values below 5 indicate that multicollinearity is not a significant problem in the model (Table 4).

**TABLE 4:** VIF statistics.

Main items	VIF
OT3	1.497
OT5	1.773
OT6	1.196
OT7	1.459
SB1	2.358
SB2	2.610
SB3	2.949
SB4	2.887
SB5	2.080
TM1	2.117
TM2	1.714
TM3	2.049
TM6	1.109

#### 4.4.3 Discriminant validity

Discriminant validity was evaluated using two commonly recommended approaches: the Fornell-Larcker criterion (Table 5) and the heterotrait-monotrait ratio (HTMT) (Table 6).

**TABLE 5:** Fornell–Larcker criterion.

Main variables	Organizational trust (OT)	Sportsmanship behavior (SB)	Trust in manager (TM)
Organizational trust (OT)	0.747	0.845	0.715
Sportsmanship behavior (SB)	0.534		
Trust in manager (TM)	0.425	0.429	

**TABLE 6:** Heterotrait–Monotrait ratio (HTMT)

Main relationships	HTMT
Trust in manager (TM) < - > organizational trust (OT)	0.509
Trust in manager (TM) < - > sportsmanship behavior (SB)	0.404
Sportsmanship behavior (SB) < - > organizational trust (OT)	0.598

According to the Fornell and Larcker criterion (Fornell and Larcker, 1981), the square root of the AVE for each construct should exceed its correlations with the other constructs in the model. As reported in Table 5, the square root of the AVE values on the diagonal are higher than the correlations between constructs. Therefore, the results provide evidence of satisfactory discriminant validity among the constructs in the measurement model (Henseler et al., 2015).

In addition, discriminant validity was further examined using the Heterotrait–Monotrait ratio (HTMT). As recommended by Henseler et al. (2015), HTMT values should be below 0.85 to establish adequate discriminant validity (see Table 6).

#### 4.4.4 Structural model assessment (hypothesis testing)

The next step was to examine the structural model to evaluate the proposed hypotheses. PLS-SEM analyzes relationships among constructs using an iterative regression procedure that simultaneously estimates both the measurement and structural models (Ringle et al., 2023). The evaluation of the structural model included examining the path coefficients (β), standard deviations, *t*-values, and *p*-values. These statistics were obtained using a bootstrapping procedure with 5,000 resamples, a commonly recommended approach in PLS-SEM analysis (Hair et al., 2019).

The results of the direct relationships are presented in Table 7. Trust in the manager had a moderate positive effect on organizational trust (β = 0.425), indicating that higher levels of trust in managers are associated with stronger perceptions of organizational trust among employees. Organizational trust also had a positive effect on sportsmanship behavior (β = 0.429), suggesting that employees who trust their organization are more likely to exhibit sportsmanship. Furthermore, trust in the manager directly influenced sportsmanship behavior (β = 0.246), indicating that managerial trust contributes to employees’ willingness to demonstrate positive discretionary workplace behaviors. The significance of these relationships was confirmed through bootstrapping (5,000 resamples), with all paths statistically significant (*p* < 0.001). These findings support the proposed hypotheses.

**TABLE 7:** Structural model results- direct relationships.

Direct relationships	*B*	Standard deviation (STDEV)	*T*-values	*P*-values
Trust in manager - > organizational trust	0.425	0.047	9.084	0.000
Organizational trust - > sportsmanship behavior	0.429	0.060	7.131	0.000
Trust in manager - > sportsmanship behavior	0.246	0.055	4.459	0.000

β, Path coefficients.

**FIGURE 2:**
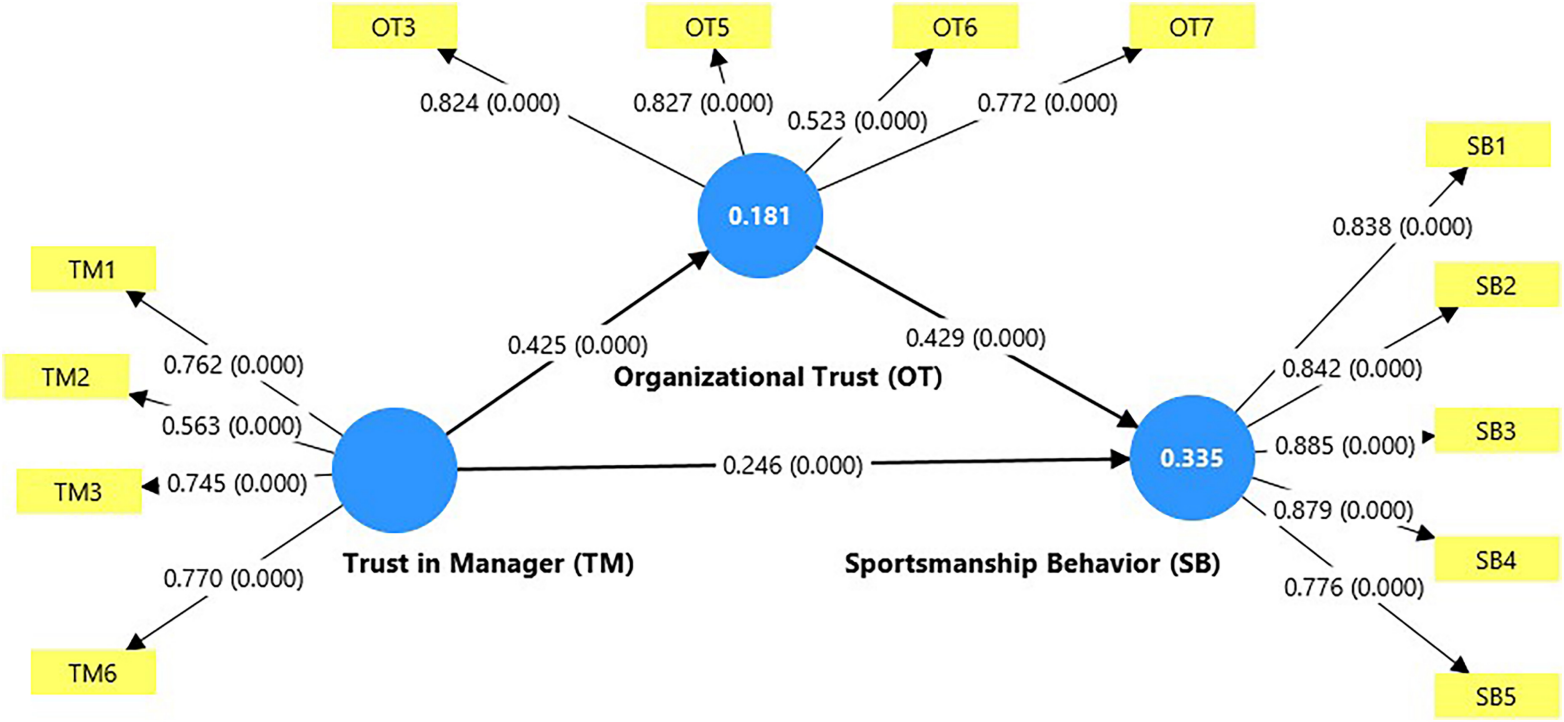
Structural model.

As shown in Table 8, the indirect relationship between trust in manager and sportsmanship behavior through organizational trust is statistically significant (β = 0.183). This finding indicates that organizational trust partially mediates the relationship between trust in manager and sportsmanship. The results of the structural model analysis are presented in Figure 2.

**TABLE 8:** Structural model results—mediation effect.

Indirect relationships	*B*	Standard deviation (STDEV)	*T*-values	*P*-values	2.5%	97.5%
Trust in Manager - > Organizational Trust - > Sportsmanship Behavior	0.183	0.035	5.217	0.000	0.123	0.261

β, Path coefficients.

#### 4.4.5 Coefficient of determination (R^2^)

The coefficient of determination (R^2^) was used to assess the model’s explanatory power. As shown in Table 9, trust in the manager explains 18.1% of the variance in organizational trust (*R*^2^ = 0.181). Furthermore, organizational trust and trust in the manager jointly explain 33.5% of the variance in sportsmanship (*R*^2^ = 0.335). According to Hair et al. (2019), these values indicate moderate explanatory power for the structural model.

**TABLE 9:** Coefficient of determination (R^2^).

Endogenous construct	R-square adjusted
Organizational trust (OT)	0.178
Sportsmanship behavior (SB)	0.331

#### 4.4.6 Effect size (f^2^)

The effect size (f^2^) was calculated to assess the contribution of each exogenous construct to the R^2^ of the endogenous constructs. As shown in Table 10, organizational trust has a medium effect on sportsmanship (*f*^2^ = 0.227). Similarly, trust in manager shows a medium effect on organizational trust (*f*^2^ = 0.221). In contrast, trust in manager shows a small effect on sportsmanship (*f*^2^ = 0.075).

**TABLE 10:** Effect size (f^2^).

Relationships	f-square
Organizational trust (OT) - > sportsmanship behavior (SB)	0.227
Trust in manager (TM) - > organizational trust (OT)	0.221
Trust in manager (TM) - > sportsmanship behavior (SB)	0.075

#### 4.4.7 Predictive relevance (Q^2^ predict)

The model’s predictive ability was evaluated using the PLS-predict procedure in Smart-PLS. The results in Table 11 show that the Q^2^ predict values for the endogenous constructs are above zero, indicating that the model has predictive capability. Specifically, the Q^2^ predict value for Organizational Trust is 0.167, and for Sportsmanship it is 0.174. According to Hair et al. (2019), Q^2^ values greater than zero indicate sufficient predictive relevance. Accordingly, these findings suggest that the model demonstrates moderate predictive power for both constructs, indicating that the exogenous variables can reasonably explain and predict variations in the endogenous variables.

**TABLE 11:** Q^2^ (predictive relevance) and the root mean square error (RMSE).

Construct	Q^2^ predict	RMSE
Organizational Trust (OT)	0.167	0.918
Sportsmanship Behavior (SB)	0.174	0.913

As reported in Table 11, the Root Mean Square Error (RMSE) values were 0.918 for Organizational Trust and 0.913 for Sportsmanship Behavior, while the SRMR value was 0.116. These indices provide supplementary information regarding the model’s predictive performance and overall fit. Consistent with the prediction-oriented nature of PLS-SEM, model evaluation primarily focused on reliability, validity, path coefficients, explanatory power (R^2^), and predictive relevance (Q^2^), while RMSE and SRMR were reported as additional assessment indicators (Hair et al., 2020; Yusefi et al., 2025).

## 5 Discussion

This study suggests that trust in one’s manager promotes sportsmanship by first building trust in the organization. Consistent with SET and LMX, when employees have positive, supportive interactions with their managers, they tend to reciprocate with constructive behaviors, including greater tolerance, cooperation, and fewer complaints.

H1 shows that trust in the manager significantly strengthens organizational trust. Employees interpret managers’ behavior—fairness, competence, and integrity—as cues about the organization’s values and intentions. Recent work finds that trust in leadership is central to developing organizational trust and positive employee attitudes (Choong et al., 2025). Trust in leadership has been shown to predict broader organizational trust in a meta-analytic study (Dirks and Ferrin, 2002). Trust in a manager (micro level) may serve as a relational gateway that shapes employees’ perceptions of the organization as a trustworthy actor (macro level) (Schilke and Lumineau, 2025). Because managers serve as visible representatives of the organization, their actions shape how employees view organizational policies and systems. Positive manager–employee relationships therefore foster confidence in the organization.

H2 is confirmed: organizational trust itself boosts sportsmanship. Prior empirical evidence further supports this relationship. For example, findings from 305 validated questionnaires collected from Chinese enterprises show that organizational trust has a significant positive influence on OCB, including cooperation and sportsmanship, indicating that employees who trust their organization are more likely to engage in voluntary behaviors that benefit the workplace (Dai et al., 2022). Employees who believe their organization treats them fairly and values their contributions feel secure and respected, making them more willing to act in ways that preserve workplace harmony.

H3 is also supported: trust in the manager positively affects sportsmanship behavior, a key form of organizational citizenship behavior (OCB). When employees trust their managers, they are more likely to respond constructively in difficult situations—showing patience, tolerating minor hassles, and avoiding unnecessary complaints. Trust reduces negative interpretations of managerial decisions and encourages cooperative responses, consistent with findings that supportive supervision directly promotes OCB. The findings align with Schaubroeck et al. (2013), who suggested that trust relationships with leaders contribute to broader organizational attachment and positive workplace outcomes through social exchange processes. Furthermore, the present findings corroborate prior evidence that relational authenticity is positively associated with altruistic and sportsmanship behaviors (Ostermeier et al., 2022). Similarly, research in leadership highlights the importance of trust in shaping specific forms of OCB, such as sportsmanship. Takos et al. (2025) found that trust in leaders directly influences employees’ sportsmanship behavior. These findings suggest that trust functions not only as a relational and emotional factor but also as a mechanism that guides employees’ behavioral responses within organizations.

Finally, the results support H4, showing that organizational trust mediates the relationship between trust in the manager and sportsmanship behavior. The findings are consistent with Mayer et al. (1995), who suggested that trust within organizational relationships facilitates positive relational outcomes and encourages employees to engage in discretionary behaviors. The significant mediating role of organizational trust indicates that trusted managerial relationships may strengthen broader trust perceptions toward the organization, which subsequently enhance sportsmanship behavior among employees. Additionally, this mediation explains how managerial behavior influences broader organizational outcomes. Specifically, trust developed through interactions with managers gradually extends to the organization as a whole. As organizational trust increases, employees become more likely to maintain positive attitudes and engage in constructive behaviors such as sportsmanship. This finding highlights trust as a dynamic relational process within organizations. Prior research also emphasizes that leadership behaviors often influence employee outcomes indirectly by shaping broader organizational perceptions that guide employee attitudes and behaviors (Choong et al., 2025; Dai et al., 2022). Additionally, the present findings are consistent with prior research showing that organizational trust functions as a key mediating variable in social exchange relationships (Liu et al., 2025). Therefore, cultivating trust at both the managerial and organizational levels is essential for encouraging positive discretionary behaviors among employees.

## 6 Practical and theoretical implications

This study offers several meaningful implications for both theory and organizational practice. From a practical perspective, the results underscore the crucial role of managerial behavior in fostering a positive work environment. When managers are perceived as fair, capable, and supportive, employees are more likely to trust them. Over time, these positive experiences with supervisors shape employees’ perceptions of the organization as a whole. As organizational trust increases, employees become more willing to tolerate workplace difficulties, reduce unnecessary complaints, and maintain constructive attitudes even in challenging circumstances. Therefore, organizations seeking to strengthen cooperation and sportsmanship among employees should focus on developing trust-based leadership practices.

Managers foster trust by making decisions openly, treating staff with respect, and acting with consistent integrity. Organizations can reinforce this by providing leadership training that emphasizes ethical behavior, clear communication, and fair management practices. By deepening trust in managers, employees are more likely to engage in voluntary, positive behaviors that enhance a cooperative and supportive workplace.

Beyond its influence on sportsmanship and other citizenship behaviors, organizational trust may have broader implications for workplace effectiveness and employee wellbeing. Prior research has shown that trust in management fosters employee cooperation, proactive citizenship behaviors, and stronger organizational relationships. Furthermore, trust is a critical factor in occupational safety, as it encourages employees to engage in safety-related citizenship behaviors, comply with organizational procedures, and contribute to a stronger safety culture. These outcomes may ultimately reduce unsafe workplace behaviors and improve organizational safety performance (Ordysiński, 2024).

From a theoretical perspective, the findings contribute to the OCB literature by identifying a relational trust mechanism that explains the emergence of sportsmanship behaviors. While prior studies have generally associated sportsmanship with broad organizational conditions or workplace environments, the present study demonstrates that trust in managers is an important antecedent that shapes employees’ broader trust in the organization. Drawing on both SET and LMX theory, the findings suggest that high-quality relationships between managers and employees foster trust, mutual respect, and positive exchanges, which extend beyond the immediate supervisor–subordinate relationship to influence employees’ perceptions of the organization. As employees develop organizational trust, they are more likely to reciprocate through sportsmanship behaviors characterized by tolerance, cooperation, and a willingness to overlook minor workplace inconveniences. Accordingly, the study extends the LMX and OCB literature by highlighting organizational trust as a key explanatory mechanism through which managerial trust is translated into positive discretionary employee behaviors.

## 7 Limitations and future research

Although this study offers useful insights into how trust influences sportsmanship behavior, several limitations should be recognized. Acknowledging these limitations clarifies the scope of the findings and offers directions for future research.

First, the proposed framework focuses primarily on trust in the manager and organizational trust as the key factors influencing sportsmanship. While these variables are strongly supported by SET and LMX, sportsmanship is likely affected by a broader set of factors. Elements such as leadership style, organizational justice, job satisfaction, psychological safety, and organizational culture may also shape employees’ willingness to tolerate difficulties and avoid unnecessary complaints. Because these variables were not included in the current model, the study captures only part of the broader explanation. Future research could incorporate additional organizational and psychological factors to develop a more comprehensive model of sportsmanship behavior.

Second, the study uses a cross-sectional research design, which limits the ability to draw strong causal conclusions. The model assumes that trust in managers gradually develops into organizational trust and then leads to sportsmanship behaviors. However, cross-sectional data capture perceptions at only one point in time and may not fully reflect how trust develops over time. Future studies could adopt longitudinal designs to better understand how trust evolves and how it influences employees’ discretionary behaviors. Additionally, this study employed a convenience sampling approach, which may limit the generalizability of the findings. Although the sample provided valuable insights into the relationships among the study variables, caution should be exercised when extending the results to all organizations in Saudi Arabia. Future research may benefit from using probability sampling techniques and examining different sectors and regions to enhance the external validity of the findings.

Third, the research relies on self-reported survey data, which may introduce biases such as common method variance or social desirability bias. Employees might unintentionally report more positive behaviors or provide responses they believe are socially acceptable. Although PLS SEM techniques can help mitigate these concerns, using a single data source remains a limitation. Future studies could improve the robustness of the findings by including multiple data sources, such as supervisor assessments or peer evaluations.

Fourth, the generalizability of the results is a limitation. Employee trust and behavior can vary across cultural, institutional, and organizational contexts. In some environments, employees may naturally display greater tolerance and cooperation, while in others they may be more likely to express dissatisfaction when facing challenges. Because this study was conducted within a specific organizational context, the findings may not fully apply to all industries or cultures. Future research should therefore test the proposed model across different countries, sectors, and organizational settings.

Fifth, the study focuses solely on sportsmanship as an aspect of OCB. Sportsmanship is crucial for maintaining a positive work environment, but OCB also includes other dimensions such as civic virtue, conscientiousness, altruism, and civility. A more comprehensive understanding of how trust influences employees’ discretionary actions may be gained by examining these additional behaviors.

Sixth, another limitation of this study concerns the sample’s gender composition, as male respondents accounted for 95.1% of participants. Although additional analyses found no significant gender differences in the key study variables, caution should be exercised when generalizing the findings to more gender-balanced workforces.

Seventh, another limitation concerns the use of self-reported data collected from a single source at a single point in time. Although Harman’s single-factor test suggested that common method bias was not a serious concern, as the first factor accounted for only 29.10% of the total variance, the possibility of common method bias cannot be entirely ruled out. Future research may benefit from collecting data from multiple sources, using longitudinal designs, or incorporating additional procedural and statistical controls to further reduce the potential effects of common method bias.

Lastly, although the measurement scales used in this study were adopted from established trust literature, more recent research has suggested broader, multidimensional operationalizations of organizational trust (Mayer and Davis, 1999; Schoorman et al., 2016). Future research may benefit from employing more contemporary trust measurement approaches that capture additional dimensions of trust relationships and further enhance construct validity.

## 8 Conclusion

Trust relationships play a foundational role in shaping positive workplace behaviors. This study integrates SET to explain how trust in the manager enhances sportsmanship through organizational trust. By strengthening trust at both the managerial and organizational levels, organizations can foster constructive, cooperative workplace climates. The findings further support the relevance of Mayer et al.’s (1995) integrative model of organizational trust in explaining how trust relationships and perceptions of trustworthiness may shape positive workplace behaviors within organizational settings.

Empirically, this study makes four contributions. First, it provides evidence that trust in managers positively influences organizational trust. Second, the findings demonstrate that trust in managers directly promotes employees’ sportsmanship. Third, the results show that organizational trust is a significant predictor of sportsmanship. Fourth, the study confirms the mediating role of organizational trust in explaining how trust in managers translates into positive discretionary employee behaviors. Collectively, these findings underscore the importance of trust-based relationships in fostering constructive workplace attitudes and behaviors.

## Data Availability

The raw data supporting the conclusions of this article will be made available by the authors, without undue reservation.

## Appendix 1

### Measurement scales used in the study

This appendix presents the measurement scales used in the study. The constructs were measured using previously validated instruments widely used in organizational behavior research. Specifically, trust in supervisor, organizational trust, and organizational citizenship behavior (OCB) were measured using established scales. In the case of OCB, only one dimension (sportsmanship) was used in the present study, as it directly reflects employees’ willingness to tolerate inconveniences and avoid unnecessary complaints at work. All items were measured using a Likert scale.

1. Trust in Manager (TM)

Trust in manager was measured using the scale developed by Podsakoff et al. (1990), which assesses employees’ level of confidence, loyalty, and faith in their immediate supervisor.

*Items*:

1. I feel quite confident that my supervisor will always try to treat me fairly.
2. My supervisor would never try to gain an advantage by deceiving workers.
3. I have complete faith in the integrity of my supervisor.
4. I feel strong loyalty to my supervisor.
5. I will support my supervisor in almost any emergency.
6. I have a divided sense of loyalty toward my supervisor. *(reverse coded)*

2. Organizational Trust (OT)

Organizational trust was measured using the scale developed by Robinson (1996), which captures employees’ perceptions of the organization’s honesty, fairness, and integrity.

*Item*s:

1. I believe my organization has high integrity.
2. I can expect my organization to treat me in a consistent and predictable fashion.
3. My organization is not always honest and truthful. *(R)*
4. In general, I believe my organization’s motives and intentions are good.
5. I do not think my organization treats me fairly. *(R)*
6. My organization is open and upfront with me.
7. I am not sure I fully trust my organization. *(R)*

3. Organizational Citizenship Behavior (OCB) – Sportsmanship Behavior

Organizational citizenship behavior was measured using the scale developed by Niehoff and Moorman (1993). The original scale includes five dimensions of OCB based on the conceptualization of Organ (1988): altruism, conscientiousness, sportsmanship, courtesy, and civic virtue. For the purposes of this study, only the sportsmanship dimension was used, as it reflects employees’ ability to maintain a positive attitude and refrain from complaining about minor workplace inconveniences.

### Sportsmanship behavior items

1. I tend to make problems bigger than they really are. *(R)*
2. I always focus on what is wrong rather than the positive side. *(R)*
3. I complain about trivial matters at work. *(R)*
4. I tend to find fault with what the organization is doing. *(R)*
5. I spend a lot of time complaining about trivial matters. *(R)*
